# Phylogenetically distinct equine influenza viruses show different tropism for the swine respiratory tract

**DOI:** 10.1099/vir.0.000049

**Published:** 2015-05

**Authors:** Livia V. Patrono, Francesco Bonfante, Claudia Zanardello, Calogero Terregino, Ilaria Capua, Pablo R. Murcia

**Affiliations:** ^1^​ Department of Animal Medicine, Production and Health, Doctoral School of Veterinary Sciences, University of Padova, Padova, Italy; ^2^​ Division of Comparative Biomedical Sciences, Istituto Zooprofilattico Sperimentale delle Venezie, Legnaro, Padova, Italy; ^3^​ Division of Specialised Diagnostics and Histopathology, Istituto Zooprofilattico Sperimentale delle Venezie, Legnaro, Padova, Italy; ^4^​ Medical Research Council-University of Glasgow Centre for Virus Research, Glasgow, UK

## Abstract

Influenza A viruses circulate in a wide range of animals. H3N8 equine influenza virus (EIV) is an avian-origin virus that has established in dogs as canine influenza virus (CIV) and has also been isolated from camels and pigs. Previous work suggests that mutations acquired during EIV evolution might have played a role in CIV emergence. Given the potential role of pigs as a source of human infections, we determined the ability of H3N8 EIVs to replicate in pig cell lines and in respiratory explants. We show that phylogenetically distinct EIVs display different infection phenotypes along the pig respiratory tract, but not in cell lines. Our results suggest that EIV displays a dynamic host range along its evolutionary history, supporting the view that evolutionary processes play important roles in host range and tropism and also underscoring the utility of using explant cultures to study influenza pathogenesis.

Influenza A viruses (IAVs) circulate in various animal hosts, and cross-species transmissions of IAVs have led to either spillover infections or emergence and sustained transmission in new host populations. Avian H3N8 viruses show a remarkable ability to cross species barriers, particularly in infecting mammals. For example, H3N8 equine influenza virus (EIV) is an avian-derived IAV (Worobey *et al.*, 2014) that was first reported in the early 1960s and is still circulating in horses. A second introduction of a different H3N8 avian influenza virus into the horse population took place in 1989 (Guo *et al.*, 1992) but this virus only circulated in equines for a couple of years (Guo *et al.*, 1995). Further, an avian-derived H3N8 IAV has recently caused an outbreak in seals in North America (Karlsson *et al.*, 2014). Finally, the currently circulating H3N8 EIV crossed the species barrier and established in dogs as canine influenza virus (CIV) in the early 2000s (Crawford *et al.*, 2005) and natural spillover infections of H3N8 EIV have been reported in pigs (Tu *et al.*, 2009) and camels (Yondon *et al.*, 2014).

Historical and epidemiological evidence support the view that pigs play an important role in influenza ecology (Vincent *et al.*, 2014) and might be a source of reassortant viruses with zoonotic potential. Therefore, it is important to determine if IAVs that are endemic in other domestic animals could potentially infect pigs and contribute via reassortment to the IAV gene pool that could eventually transfer to humans, as happened during the 2009 pandemic (Smith *et al.*, 2009).

It has been suggested that EIV evolution could have played an important role in the emergence of CIV: EIVs that circulated in the 1960s displayed a highly attenuated phenotype in dog tracheas, whereas an EIV isolated in 2003 – around the time of emergence of canine influenza virus – exhibited an infection phenotype indistinguishable from that of CIV (Gonzalez *et al.*, 2014).

We wanted to investigate whether changes in host range along EIV evolutionary history included other animal species besides dogs, and focused on pigs because this species can behave as a ‘mixing vessel’ (Ma *et al.*, 2009) and also because EIV has been isolated from pigs in nature (Tu *et al.*, 2009). To this end, we inoculated two different swine cell lines (newborn pig trachea cells and newborn swine kidney cells) with a Eurasian H3N2 swine influenza virus A/swine/Italy/8088/2006 (SIV) and compared its growth kinetics to those of a panel of H3N8 EIVs: A/equine/Uruguay/1963 (Uruguay/63), A/equine/Fontainebleau/1979 (Fontainebleau/79), A/equine/Argentina/1995 (Argentina/95) and A/equine/South Africa/2003 (South Africa/2003). These viruses represent phylogenetically distinct clades of EIV and despite the unavailability of complete passage history they were chosen for specific reasons: Uruguay/63 is the oldest known H3N8 EIV (Murcia *et al.*, 2011), South Africa/03 represents the clade of viruses circulating at the time of CIV emergence and the other two isolates included represent intermediate clades. The swine cell lines (provided by the Istituto Zooprofilattico Sperimentale della Lombardia ed Emilia Romagna; Ferrari *et al.*, 2003) were maintained at 37 °C with 5 % CO_2_ in minimum essential medium (MEM; Gibco) supplemented with 10 % FCS (Euroclone), 1 % 200 mM l-glutamine (Sigma) and 1 % penicillin-streptomycin (P/S; Gibco) and nystatin (Sigma). In this first study we compared the growth kinetics of the different viruses over a 72 h time-course (m.o.i. 0.001). Viral titres in the supernatants from triplicate wells of three independent experiments were calculated by the TCID_50_ assay according to the Reed and Muench formula. Briefly, samples were serially diluted in MEM supplemented with 1 % P/S, 1 % l-glutamine and 1 µg TPCK-treated trypsin ml^−1^ (infection medium) and 50 µl of each dilution was inoculated in quadruplicate wells of confluent MDCK cells seeded in 96-well plates. After a two-hour incubation at 37 °C, 100 µl of infection medium was added to each well and plates were further incubated for 72 h. Plates were checked for the presence of CPE and immunostained as described previously (Gonzalez *et al.*, 2014). Although there were slight differences in replication dynamics observed at 6 and 24 h post-infection (p.i.), no significant differences were detected between SIV and any of the EIVs tested at later time points (*P*>0.05 two-way ANOVA with Bonferroni’s post-test for multiple comparisons). These results show that all tested EIVs can readily infect and replicate in swine cell lines to similar levels of an H3N2 swine influenza virus (Fig. 1a, b).

**Fig. 1.**
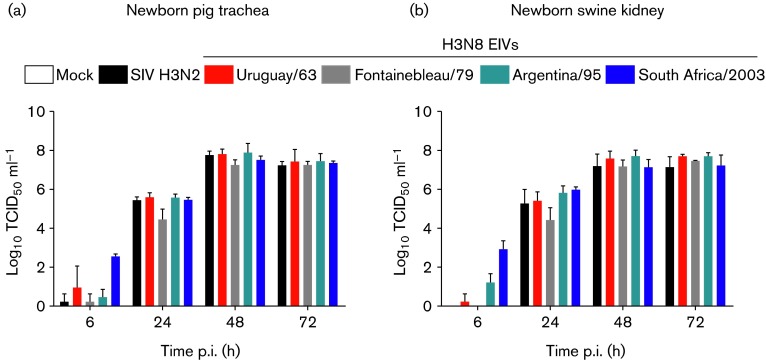
Growth kinetics of evolutionary distinct H3N8 EIVs in swine cell lines. (a) Newborn pig trachea cells. (b) Newborn swine kidney cells. Vertical bars show the mean and sd of three independent experiments.

Since influenza in pigs is a respiratory disease we then sought to characterize the infection phenotypes of the different EIVs in the swine respiratory tract. Tissue explants represent a suitable model to study influenza pathogenesis at the site of infection and also allow for a reduction of the number of animals used in research. Experiments were approved by the ethical committee of IZSVe (Approval number CE IZSVe 11_2012). We cultured explants from various anatomical regions of the swine respiratory tract: nasal mucosa (respiratory section), trachea (all tracheal rings were used) and lung (right apical lobe). Tissues were harvested from six- to eight-week-old commercial hybrid piglets that were IAV seronegative. Explants were prepared as previously described (Nunes *et al.*, 2010; Van Poucke *et al.*, 2010), and inoculated with 200 p.f.u. of SIV H3N2, or with the aforementioned EIVs. Infection phenotypes were determined at 6, 24, 48, 72 and 96 h post-inoculation based on virus growth kinetics (TCID_50_ ml^−1^ as described above), histopathological lesions, changes in ciliary beating and viral antigen detection by immunohistochemistry as described in Gonzalez *et al.*, (2014). Each virus was tested in duplicate in tissues obtained from three different pigs.

As expected, SIV consistently exhibited high replication efficiency in tracheas (Fig. 2b), lungs (Fig. 3b) and nasal mucosa (not shown). Histological damage was evident in the nasal mucosa displaying lesions such as epithelial disruption and vacuolization (Fig. 2a). Infected tracheas exhibited loss of cilia, reduction in epithelial thickness (Fig. 2d) and decreased ciliary function (Fig. 2c). SIV H3N2 antigen was also readily detected in all infected explants regardless of the anatomical location (Figs 2a, d and 3a).

**Fig. 2.**
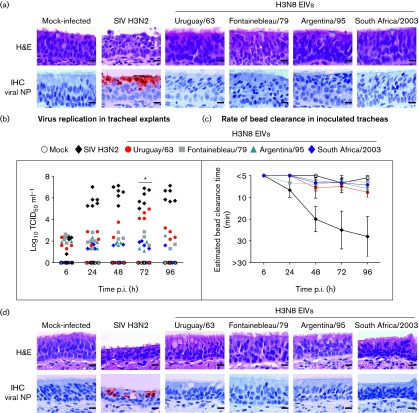
Infection of explants derived from swine nasal mucosa and trachea with evolutionary distinct H3N8 EIVs. (a) Histological features of swine nasal mucosa explants infected with H3N2 SIV (positive control) and various H3N8 EIVs (Uruguay/63, Fontainebleau/79, Argentina/95 and South Africa/2003). Lesions are shown in sections stained with haematoxylin and eosin (H&E). Infected cells were detected by immunohistochemical (IHC) staining of the NP viral protein. Positive cells are stained in brown. Black horizontal bars represent 50 µm. (b) Growth kinetics of the viruses described in (a) in swine tracheal explants. Dots represent values of individual replicates, * *P*<0.05. (c) Graphical representation of bead clearance assays in infected and control swine tracheal explants. Lines represent the average time to clear the beads in three independent experiments. Error bars represent SEM. (d) Histological features of swine tracheal explants infected with the viruses described in (a). Lesions and infected cells are described as in (a). Bars, 50 µm. Panels (a) and (c) show explants at day two p.i.

**Fig. 3.**
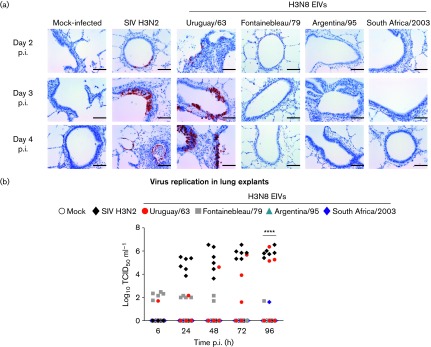
Uruguay/63 replicates in swine lung explants at higher levels than other EIVs. (a) Immunohistochemical detection of viral nucleoprotein in lung explants infected with the same viruses as in Figs 1 and 2. Positive cells are stained brown. Bars, 50 µm. (b) Growth kinetics of the viruses in swine lung explants. Dots represent values of individual replicates, ****P<0.0001.

In contrast, EIVs showed variable infection phenotypes depending on the virus isolate and the anatomical region of the respiratory tract tested. The nasal mucosa was the only anatomical region in which no signs of EIV infection and replication were observed. In the trachea, only one of the four EIVs tested (Uruguay/63) replicated at higher titres than the other EIVs, but still at lower levels than SIV (Fig. 2b). Uruguay/63 exhibited a peak in viral replication (up to 5 log_10_ TCID_50_ ml^−1^) at 72 h p.i. in four out of six replicas. As for the other EIVs, virus titres were always lower or equal to the initial inoculum suggesting that they replicated at very low levels. The differences observed between Uruguay/63 and the other EIVs were significant at 72 h p.i. (*P*<0.05 two-way ANOVA with Bonferroni’s post-test for multiple comparisons). However, despite evidence of viral replication, Uruguay/63 did not cause histological changes or alterations in bead clearance (Fig. 2c, d, respectively). Further, no viral antigen was detected in the infected tracheas examined despite extensive serial sectioning of infected explants (Fig. 2d). The absence of lesions in explants infected with IAVs that replicate at high levels has been previously described (Gonzalez *et al.*, 2014; Van Poucke *et al.*, 2010). Focal infections in limited areas of the explants have been suggested to be the cause of such disparity (Chan *et al.*, 2013).

In lung explants the difference in infection phenotypes was more evident: Uruguay/63 displayed higher replication efficiency by day two post-inoculation, with up to four log increases and peaking at day four, although some inter-animal differences in replication dynamics were observed (Fig. 3b). In fact, in some explants viral titres of Uruguay/63 at 96 h p.i. were similar to those exhibited by SIV H3N2 and significantly higher than those observed for the other EIVs (*P*<0.0001 two-way ANOVA with Bonferroni’s post-test for multiple comparisons). Moreover, based on immunohistochemical detection of the viral nucleoprotein, we observed that Uruguay/63 infected the bronchiolar epithelium and rare alveolar cells at comparable levels to SIV H3N2 (Fig. 3a). The other EIVs tested did not replicate (or replicated at very low levels) in infected lung explants and no viral antigen was detected by immunohistochemistry despite testing multiple serial sections.

Overall, our results indicate that despite the ability of all tested EIVs to efficiently replicate in pig cell lines in monolayer cultures – in fact, to similar levels of an H3N2 swine influenza virus – only Uruguay/63 is able to infect and replicate efficiently in distinct anatomical regions of the pig respiratory tract. Explants infected with H3N2 SIV exhibited consistent results in all tested replicas (Figs 2b and 3b). In contrast, tissues inoculated with EIVs showed some variability in infection despite taking precautions to minimize experimental variability (e.g. by infecting the same tracheal rings of different animals with the same virus, and exclusively using the right apical lobe of the lung). Similar variations in infection phenotypes have been reported in swine tracheal and lung explants infected with human isolates of H5N1 avian influenza virus (Chan *et al.*, 2013). Although the cause of those variable results has not been determined, it has been suggested that they could be due to variation in receptor abundance and distribution among explants, as well as variable levels of mucus (in the trachea) and surfactant protein D (in the lungs). All these factors could play important roles in determining the efficiency of influenza virus infection and thus should be taken into account (Hillaire *et al.*, 2013; Matrosovich & Klenk, 2003). Further studies addressing the quantification and variability of such factors in *ex vivo* systems could be important to clarify their potential influence on the experimental variation observed.

Here we show that Uruguay/63, the oldest EIV isolate, can infect swine tracheal (albeit with some variability) and lung explants but not the nasal mucosa. Notably, lungs infected with Uruguay/63 showed similar levels of infection to those observed with SIV H3N2, a swine-adapted influenza virus. In contrast, all the other EIVs tested showed an impaired ability to infect any portion of the pig respiratory tract. As previous reports indicate that the swine respiratory tract supports infection and replication of some avian influenza viruses (Löndt *et al.*, 2012; Van Poucke *et al.*, 2010), it is not surprising that Uruguay/63 – the most avian-like virus of the H3N8 EIV lineage – exhibited the highest ability to replicate in pig respiratory tissues. It is feasible to think that the initial EIVs were more ‘avian-like’ and thus able to infect pigs, and that such tropism for the swine respiratory tract was lost when EIV became more adapted to the horse during the initial EIV epidemics. Then, EIV acquired the ability to infect dogs through genetic drift during continuous evolution in horses. Notably, Uruguay/63 and South Africa/2003 displayed very different infection phenotypes in the dog tracheas: South Africa/2003 infects dog tracheas in a similar to fashion that of canine influenza viruses, whereas Uruguay/63 is highly attenuated (Gonzalez *et al.*, 2014) supporting the view that evolutionary processes result in adaptive changes in the viruses that impact both on host range and viral tropism.

The finding that all EIVs tested replicated at similar levels to an H3N2 swine influenza virus in pig cell lines, whereas distinct infection phenotypes were observed in explants, suggests the presence of tissue-specific host barriers at the site of infection that must play a central role in viral pathogenesis and emergence, and highlights the importance of using relevant biological systems to assess changes in host range.

Our study has various limitations. First, we tested a small number of EIVs, albeit the choice of viruses was based on a previous study that showed an association between the infection phenotype of phylogenetically distinct EIVs in dog tracheas and the emergence of CIV (Gonzalez *et al.*, 2014). Here we included the same viruses used in that study and also tested other EIV isolates (Fontainebleau/79 and Argentina/95), which represent additional phylogenetically distinct clades. Second, while our results do not necessarily mean that EIV will or could have emerged in pigs, they do not rule out that possibility either. Previous reports on the correlation of *ex vivo* and *in vivo* infections (Van Poucke *et al.*, 2010), together with the isolation of EIV in pigs in Asia (Tu *et al.*, 2009), indicates that natural infection of EIV in swine can (and did) occur. Given the important role of the pig as a contributor to the gene pool of human influenza viruses it is important to determine if other IAVs could expand that gene pool via infections in swine. Thus, identifying the risk of emergence of currently circulating viruses (or their genes) is important from the point of view of pandemic preparedness. Third, we did not attempt to identify the genetic determinants that allow EIV to productively infect the respiratory tract of the pig and therefore our study could be considered observational. Future studies using reassortant and mutant viruses generated by reverse genetics will be required to achieve that task.

In conclusion, we showed that a phylogenetically distinct EIV displays an enhanced tropism for the respiratory tract of the pig compared to other viruses of the same lineage. Our results support the hypothesis that viral evolution during long-term transmission of influenza viruses in host populations could result in dynamic changes in their host range. Such changes must be in line with ecological and epidemiological factors in order to allow the establishment of novel lineages in susceptible species.
